# Supplementary material for: Role of Lipocalin-2 in Amyloid-Beta Oligomer-Induced Mouse Model of Alzheimer’s Disease
Source: Antioxidants (Basel). 2021 Oct 21;10(11):1657. doi: 10.3390/antiox10111657 (PMC8614967; doi:10.3390/antiox10111657)
Supplement: Supplementary file 1 [file antioxidants-10-01657-s001.zip › antioxidants-1420954-supplementary-done.pdf]

antioxidants

MDPI

Table S1. List of primary antibodies.

Antibody
LCN2
β-Amyloid
p-Tau (Ser396)
Iba-1

IL-6
TNF-α
HMGB1
TLR4
RAGE
NF-κBp65

GFAP
Ly6G
eNOS
ZO-1
VCAM-1

Albumin	
LCN2	
MMP-9	
p-STAT3 (Tyr705)	
STAT3	

HO-1	
Ferritin	
Ceruloplasmin	
β-actin	

IF, immunofluorescence; IHC, immunohistochemistry; WB, western blot
